# Meio Ambiente e o Coração

**DOI:** 10.36660/abc.20230119

**Published:** 2023-06-28

**Authors:** Antonio Eduardo Monteiro de Almeida, Ricardo Stein

**Affiliations:** 1 Cardio Lógica Ltda João Pessoa PB Brasil Cardio Lógica Ltda, João Pessoa, PB – Brasil; 2 Universidade Federal do Rio Grande do Sul Porto Alegre RS Brasil Universidade Federal do Rio Grande do Sul, Porto Alegre, RS – Brasil

**Keywords:** Meio Ambiente, Equilíbrio Ecológico/métodos, Poluição Ambiental, Prevenção e Controle, Estresse Oxidativo, Doenças Cardiovasculares

Nosso planeta está enfrentando uma crise sem precedentes relacionada às mudanças climáticas e à perda de biodiversidade, evidências essas que têm ameaçado a saúde dos nossos ecossistemas, comunidades, assim como modificado o estilo de vida dos seres que vivem na terra. Por sinal, muitos cientistas têm afirmado que “temos anos e não décadas para combater essa crise”.

Desde a escola primária aprendemos sobre a importância do oxigênio para vida humana. Estima-se que 50 a 80% do oxigênio da Terra seja produzida nos oceanos (“Pulmões do Planeta”), além de ser o maior meio de absorção do carbono, desempenhando papel fundamental na regulação do clima global.^1 , 2^ As florestas e demais ecossistemas produzem o restante de oxigênio que utilizamos. Indo de encontro ao binômio produção de oxigênio-absorção do carbono, a destruição destes ambientes eleva o grau de poluição e as consequências são graves e podem ser ainda mais nefastas.

A poluição do ar é uma mistura complexa e dinâmica de numerosos compostos na forma gasosa, sendo partículas proveniente de várias fontes, as quais estão sujeitas à transformação, variando no espaço e no tempo. As substâncias mais agressivas ao ser humano estão sob a forma de Partículas Totais em Suspensão (PTS), Fumaça e Partículas Inaláveis (PM_10µg_ e PM_2,5 µg_), Ozônio (O_3_), Dióxido de Nitrogênio (NO_2_), Dióxido de Enxofre (SO_2_), Monóxido de Carbono (CO) e metais como Arsênio, Chumbo, Cádmio, Mercúrio e Cobre. Em síntese, as vias fisiopatológicas dominantes incluem a ativação do estresse oxidativo - inflamação e desequilíbrio autonômico, bem como translocação de componentes da mistura de PM na circulação sistêmica.^3^ Por sua vez, essas alterações podem promover doença cardiovascular (DCV) subclínicas (remodelação miocárdica, progressão da aterosclerose, hipertensão arterial sistêmica e pulmonar, aumento da vasoconstrição e coagulação), além da DCV aguda trombótica e não trombótica (síndromes coronárias agudas, insuficiência cardíaca, acidente vascular cerebral e arritmias de alto risco).^4 - 8^

Neste cenário onde o equilíbrio é tão relevante, a poluição do ar é um fator de risco para que as DCV incidam, sendo ela um dos principais contribuintes para a carga global de doenças.^2^ Além disso, a poluição ambiental contribui para comorbidades que pioram o prognóstico entre pessoas infectadas com o SARS-Cov 19 (vírus da COVID-19), dado este proveniente de uma declaração conjunta divulgada em 28 de janeiro de 2021 por quatro das maiores instituições científicas de cardiologia do mundo: “American College of Cardiology, World Heart Federation (WHF), American Heart Association (AHA) e European Society of Cardiology (ESC)”.^3^

Segundo a Organização Mundial de Saúde (OMS)^4^ sete milhões de pessoas morrem a cada ano por causa da poluição, sendo 25% por doença cardíaca e 24% por acidente vascular cerebral (AVC). Ainda, de acordo com dados da OMS, nove em dez pessoas moram em locais onde a poluição do ar externa excedem os limites recomendados. Por sua vez, a poluição do ar interno das casas afeta em torno de três bilhões de pessoas, já que fogões movidos a querosene ou combustíveis sólidos são utilizados. Nestas situações, a fumaça interna (poluição doméstica) é 100 vezes mais alta do que os níveis recomendados e, neste cenário, 3,8 milhões de pessoas morrem anualmente, sendo 45% por doença cardíaca ou AVC.^8^

A exposição à poluição do ar relacionada ao tráfego viário pode contribuir para o aumento da prevalência de hipertensão arterial (HAS) para moradores de bairros próximos a rodovias. Hudda et al.^9^ realizaram ensaio clínico randomizado-cruzado, em ambiente controlado com monitoramento do número de partículas e concentração do carbono negro, mostrando o efeito agudo do uso de filtros na redução da pressão arterial sistólica (PAS). O aumento da PAS foi relacionado à magnitude de exposição sendo de 0,6 mmHg para baixa exposição, 1,3 mmHg para média exposição e 2,8 mmHg para alta exposição. Lanyu et al.,^10^ através de uma metanálise, evidenciaram que o uso de combustível sólido doméstico foi significativamente associado a um risco aumentado de hipertensão. Qin et al.,^11^ mostraram que o risco de hipertensão em adultos estava significativamente associado com aumento de cada 10 μg/m^3^ na exposição ambiental à longo prazo.

Por sua vez, a exposição profissional à poluição está associada a arritmias cardíacas. Vanchiere et al.,^12^ mostraram que os bombeiros que combateram um maior número de incêndios por ano tiveram maior prevalência de fibrilação atrial (OR- *Odds Ratio-* 1,14; p=0,006), assim como Wang et al.,^13^ publicaram uma metánálise relatando que a exposição ao PM_2,5_ foi significativamente relacionada ao aumento da incidência de FA em idosos. Tanto o OR combinado quanto o valor do % de alteração foram maiores em áreas com níveis mais altos de PM_2,5 µg_ (≥25 μg/m^3^ ).

A exposição por longo tempo à poluição do ar externa está associada a doença arterial coronariana (DAC)^14^ e ao AVC.^15^ Em uma importante análise agrupada de dados individuais de seis estudos de coorte do Estudo ELAPSE,^16^ os quais tiveram um acompanhamento médio de 17,2 anos, a exposição prolongada à poluição do ar foi associada à incidência de AVC (taxa de aumento no risco de 10% para cada 5 μg/m^3^ de aumento no PM_2,5 e_ 8% para cada 10 µg/m^3^ de aumento no NO_2_. Já para incidência de DAC a associação ocorreu apenas com o NO_2_, mesmo em concentrações de poluentes inferiores aos valores-limite atuais estabelecidos pelos órgãos fiscalizadores. O Estudo KPNC,^17^ realizado no norte da Califórnia, avaliou a associação da poluição por PM_2,5_ e mortalidade por DCV, AVC e infarto do miocárdio (IAM). Observou-se um aumento na mortalidade por DCV ( *Hazard Ratio-* HR-, 2,31; 95% IC, 1,96-2,71), AVC (HR, 1,41; 95% IC, 1,09-1,83]) e IAM (HR, 1,51; IC 95%, 1,21-1,89) para cada aumento de 10 µg/m^3^ na média do PM_2,5_ em 1 ano, mesmo em níveis de exposição <12 µg/m^3^ . Aliás, o aumento dos riscos observados em níveis de exposição <12 µg/m3 destaca que os atuais níveis de regulamentação PM_2,5_ podem não ser protetores para essa população suscetível.

Poluição do ar associado ao risco em curto prazo de IAM com supradesnivelamento do segmento ST (STEMI) foi analisado em investigação cruzada realizada pelo grupo do SWEDEHEART.^18^ A conclusão deste experimento foi que o risco de STEMI aumenta horas após a exposição a poluentes atmosféricos, com maior impacto para a ação do NO_2_. Através de metanálise, Lee et al.,^19^ sugeriram que a razão de risco agrupada para IAM foi de 1,052 (95% IC 1,017-1,089) para cada aumento de 1 mg/m^3^ na concentração de CO ambiente. No entanto, estudos mais robustos são necessários para confirmar a associação da poluição do ar com o IAM de fato.

Evidências que examinaram a associação entre a poluição do ar e a descompensação aguda por insuficiência cardíaca^20^ mostraram que a hospitalização ou morte estava relacionada a aumento no CO (3,52% por uma parte por milhão; 95% IC 2,52-4,54), SO_2_ (2,36% por 10 partes por bilhão; IC 1,35-3,38) e NO_2_ (1,70% por 10 partes por bilhão; IC 1,25-2,16). Já, as concentrações de partículas estavam associadas com hospitalização por insuficiência cardíaca ou morte (PM_2,5_ 2,12% por 10 μg/m^3^ , IC 95% 1,42-2,82; PM_10_: 1,63% por 10 μg/m^3^ , 95% IC 1,20-2,07). A saber, elas eram tanto mais fortes ao serem observadas no dia de exposição, sendo os efeitos mais persistentes para PM_2,5_.

A relação entre a exposição materna à poluição do ar e o risco de doença congênita cardíaca na prole também tem sido estudada. Em metanálise realizada por Hu et al.,^21^ a exposição ao CO foi associada a um risco aumentado de tetralogia de Fallot (OR 1,21, IC 95% 1,04-1,41]. Elevação do risco de comunicação interatrial (CIA) foi encontrado (para cada 10 mg/m^3^ de aumento), assim como quando ocorria incremento de 10 ppb no PM_10_ e exposição ao O_3_, respectivamente (OR 1,04, 95% IC 1,00 - 1,09; e OR 1,09, 95% IC 1,02 - 1,17). Por sua vez, a exposição categórica ao NO_2_ foi associada um risco aumentado de coarctação da aorta (OR para alto versus baixo 1,14, IC 95% 1,02 – 1,26).

Chowdhury et al.,^22^ ao avaliarem a associação da poluição por metais e desfechos cardiovasculares mostraram que a exposição ao arsênico, chumbo, cádmio e cobre estavam associados a um risco aumentado de DCV, em especial à DAC. Houve uma relação linear de dose resposta para arsênico, chumbo e cádmio com desfechos cardiovasculares.

O estudo PURE^23^ avaliou a associação de 14 possíveis fatores de risco modificáveis com mortalidade cardiovascular, tendo sido realizado em 21 países de 5 continentes, observados níveis de escolaridade e renda. A poluição do ar esteve associada a uma maior proporção de DCV e de mortes em países de baixa renda. Já naqueles de renda baixa e média, a poluição do ar doméstico, dieta inadequada e baixa escolaridade tiveram um efeito mais forte sobre a morte cardiovascular do que em países de alta renda. De acordo com o PURE, a ideia que fica é de uma lógica perversa: os países ricos poluem e os pobres adoecem e morrem.

Uma informação importante a ser discutida diz respeito ao impacto de uma possível redução na mortalidade global atribuída à poluição. Dados da OMS descrevem que a poluição por exposição anual a níveis de PM_2,5_ de 10 µg/m^3^ , se aplicada a nova recomendação que consta na Diretriz 2021 da OMS^24^ para níveis permissíveis de exposição a PM_2.5_ de 5 µg/m^3^ , a mortalidade poderia ser reduzida em 79,5%. Cabe salientar que em 2019, segundo o *Institute for Health Metrics and Evaluation* , a poluição do ar ocupou a quarta posição como fator de risco modificável, ficando atrás apenas a hipertensão arterial, uso de tabaco e dieta inapropriada.^3^

Temos ainda as alterações climáticas extremas (enchentes, incêndios e grandes áreas de degelo), as quais também se relacionam de forma direta com a poluição, além de outros fatores que aumentam o aquecimento global, impactado direta e/ou indiretamente na saúde global do planeta. Essas mudanças estão acontecendo com um aquecimento médio de apenas 1,1°C em relação aos níveis pré-industriais. O mais recente relatório do Painel Intergovernamental sobre Mudanças Climáticas (IPCC)^25^ conclui que isso é apenas uma amostra do que está por vir. Esse relatório mostra que o mundo provavelmente atingirá ou excederá 1,5 °C de aquecimento nas próximas duas décadas – mais cedo do se imaginava em avaliações anteriores. Logo, limitar o aquecimento a este nível e evitar os impactos climáticos mais severos depende de ações ainda nesta década. Somente cortes ambiciosos nas emissões permitirão manter o aumento da temperatura global em 1,5°C, o limite que os cientistas dizem ser necessário para prevenir impactos climáticos de maior monta. Em um cenário de altas emissões, o IPCC constata que o mundo pode aquecer muito mais, atingindo os apocalípticos 5,7°C até 2100.

**Figura 1:**
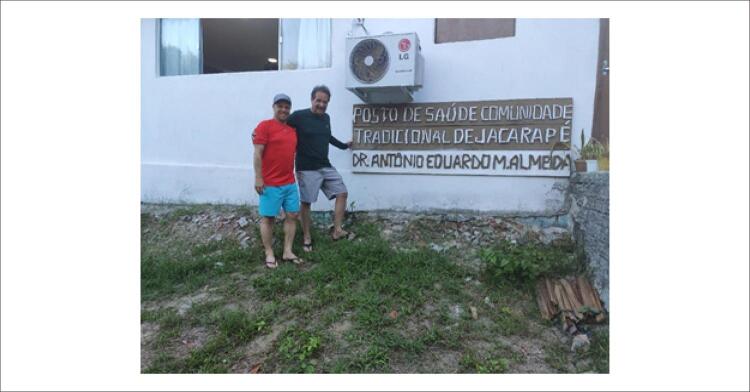
– Posto de saúde na pequena e tradicional comunidade de Jacarapé (PB).

Já quase finalizando e mudando um pouco o tom, saindo do destrutivo e indo para o construtivo. No nordeste do Brasil e certamente em muitos outros lugares do nosso país, existem movimentos em prol da saúde da natureza. E é isso que está acontecendo na pequena e tradicional comunidade de Jacarapé, praia paraibana. A partir da iniciativa do primeiro autor deste artigo em parceria com diversos membros da comunidade, práticas de educação ambiental vem sendo desenvolvidas a pleno. Tendo como base o pequeno Posto de Saúde ( Figura 1 ), a relação entre poluição do meio ambiente e risco cardiovascular tem sido amplamente discutida. Através de palestras com pacientes, outros membros da comunidade, grupos escolares da região, assim como com visitantes, a educação quanto a poluição ambiental é realizada. Além disso, em Jacarapé está sendo construído um Observatório Ambiental, este em conjunto com a Universidade Federal da Paraíba (UFPB). Ali, políticas sustentáveis de saneamento básico biológico, como também o replantio de 5000 mudas de manguezal, além do monitoramento da qualidade das águas dos rios Jacarapé e Cuiá, estão sendo realizados. Estas práticas, nas suas devidas proporções do microcosmo, estão alinhadas com a fala de Richard J. Kovacs, ex-presidente do *American College of Cardiology.* ^3^ “Os médicos têm a responsabilidade de educar seus pacientes, seus colegas e suas comunidades em geral sobre a conexão entre a poluição do ar e o risco de DCVs”.

Por fim, para enfrentar este cenário de degradação exponencial do meio ambiente, também cabe a nós cardiologistas convocar a população em geral e, principalmente os trabalhadores da área de saúde, para ações que reduzam de forma drástica a destruição da natureza. Atuar contra a poluição é antes de mais nada um ato de cidadania, ato este apartidário e em prol dos que votam nos mais diferentes partidos ou que torcem para os distintos clubes de futebol Brasil afora, ou seja, todos nós. Logo, para ir de encontro à degradação exponencial do lugar onde vivem mais de 8 bilhões de habitantes (entre outros tantos seres vivos), a frase do poeta paraibano Geraldo Vandré *cai como uma luva* : “Quem sabe faz a hora não espera acontecer”.
