# Supplementary material for: Meio Ambiente e o Coração
Source: Arq Bras Cardiol. 2023 Jun 28;120(7):e20230119. [Article in Portuguese] doi: 10.36660/abc.20230119 (PMC10365009; doi:10.36660/abc.20230119)
Supplement: Translation

# Translation: The Environment and the Heart

**Authors:** Antonio Eduardo Monteiro de Almeida, Ricardo Stein

**Affiliations:** 1 Brazil Cardio Lógica Ltda,João Pessoa, PB – Brazil; 2 Brazil Universidade Federal do Rio Grande do Sul, Porto Alegre, RS – Brazil

**Keywords:** Environment, Ecological Equilibrium/methods, Environmental, Pollution, Prevention and Control, Oxidative Stress, Cardiovascular Diseases

Our planet is facing an unprecedented crisis related to climate change and the loss of biodiversity, and there is evidence that this crisis has threatened the health of our ecosystems and communities, in addition to modifying the lifestyle of living beings on Earth. Moreover, multiple scientists have stated that “we have years rather than decades to fight this crisis.”

Since elementary school, we learn about the importance of oxygen for human life. It is estimated that 50% to 80% of the oxygen on Earth is produced in the oceans (“the planet’s lungs”), which is also the largest means of absorbing carbon, playing a fundamental role in regulating the global climate.^1 , 2^ Forests and other ecosystems produce the rest of the oxygen we use. Going against the dichotomy of producing oxygen and absorbing carbon, the destruction of these environments increases the degree of pollution; the consequences are severe and can be even more detrimental.

Air pollution is a complex and dynamic mixture of numerous compounds in gaseous form, comprising particles from various sources, which are subject to transformation, varying in space and time. The most aggressive substances to humans are in the form of total suspended particles, smoke and inhalable particles (PM_10_and PM_2.5_), ozone (O_3_), nitrogen dioxide (NO_2_), sulfur dioxide (SO_2_), carbon monoxide (CO), and metals such as arsenic, lead, cadmium, mercury, and copper. In summary, the dominant pathophysiological pathways include activation of oxidative stress, inflammation and autonomic imbalance, as well as translocation of components of the PM mixture into the systemic circulation.^3^ In turn, these alterations can promote subclinical cardiovascular disease (CVD) (myocardial remodeling , progression of atherosclerosis, systemic and pulmonary arterial hypertension, increased vasoconstriction, and coagulation), in addition to acute thrombotic and non-thrombotic CVD (acute coronary syndromes, heart failure, stroke, and high-risk arrhythmias).^4 - 8^

In this scenario, where equilibrium is so relevant, air pollution is a risk factor for CVD to occur, and it is one of the main contributors to the global burden of disease.^2^ Furthermore, environmental pollution contributes to comorbidities that worsen prognosis among people infected with SARS-CoV-2 (the virus that causes COVID-19), according to data from a joint statement released on January 28, 2021 by 4 of the largest cardiology institutions worldwide: American College of Cardiology, World Heart Federation, American Heart Association, and European Society of Cardiology.^3^

According to the World Health Organization (WHO),^4^ 7 million people die each year due to pollution, 25% of them from heart disease and 24% from stroke. Also according to WHO data, 9 out of 10 people live in places where outdoor air pollution exceeds recommended limits. In turn, indoor air pollution in homes affects around 3 billion people, where stoves powered by kerosene or solid fuels are used. In these situations, indoor smoke (domestic pollution) is 100 times higher than recommended levels and, in this scenario, 3.8 million people die annually, 45% of them from heart disease or stroke.^8^

Exposure to air pollution related to road traffic may contribute to increased prevalence of arterial hypertension in residents of neighborhoods close to highways. Hudda et al.^9^ carried out a randomized crossover clinical trial, in a controlled environment with monitoring the number of particles and black carbon concentration, showing the acute effect of using filters in reducing systolic blood pressure. The increase in systolic blood pressure was related to the magnitude of exposure, as follows: 0.6 mmHg for low exposure, 1.3 mmHg for medium exposure, and 2.8 mmHg for high exposure. A meta-analysis by Lanyu et al.^10^ provided evidence that the use of domestic solid fuel was significantly associated with an increased risk of hypertension. Qin et al.^11^ showed that the risk of hypertension in adults was significantly associated with each 10-μg/m^3^ increase in long-term environmental exposure.

In turn, occupational exposure to pollution is associated with cardiac arrhythmias. Vanchiere et al.^12^ showed that firefighters who fought a greater number of fires per year had a higher prevalence of atrial fibrillation (odds ratio [OR] 1.14; p = 0.006), and Wang et al.^13^ published a meta-analysis reporting that exposure to PM_2.5_was significantly related to increased incidence of atrial fibrillation in elderly patients. Both the combined OR and the percentage change were higher in areas with higher levels of PM_2.5_(≥ 25 µg/m^3^ ).

Long-term exposure to outdoor air pollution is associated with coronary artery disease^14^ and stroke.^15^ In an important pooled analysis of individual data from 6 cohort studies from the ELAPSE Study,^16^ which had a median follow-up of 17.2 years, prolonged exposure to air pollution was associated with stroke incidence, with an increase rate of 10% for each 5-µg/m^3^ increase in PM_2.5_and 8% for each 10-µg/m^3^ increase in NO_2_. Regarding the incidence of coronary artery disease, the association occurred only with NO_2_, even in concentrations of pollutants lower than the current limit values established by the inspection agencies. The KPNC Study,^17^ carried out in Northern California, evaluated the association of PM_2.5_pollution and mortality from CVD, stroke, and myocardial infarction. They observed an increase in mortality from CVD (hazard ratio [HR] 2.31; 95% confidence interval [CI] 1.96 to 2.71), stroke (HR 1.41; 95% CI 1.09 to 1.83) and myocardial infarction (HR 1.51; 95% CI 1.21 to 1.89) for each 10-µg/m^3^ increase in mean PM_2.5_in 1 year, even at exposure levels < 12 µg/m^3^ . The increased risks observed at exposure levels < 12 µg/m_3_highlight that current PM_2.5_regulatory levels may, in fact, not be protective for this susceptible population.

Air pollution associated with short-term risk of ST-segment elevation myocardial infarction (STEMI) was analyzed in a case-crossover study by the SWEDEHEART group.^18^ The conclusion of this experiment was that the risk of STEMI increases hours after exposure to air pollutants, with greater impact due to the action of NO_2_. Through meta-analysis, Lee et al.^19^ suggested that the pooled risk ratio for myocardial infarction was 1.052 (95% CI 1.017 to 1.089) for each 1-mg/m^3^ increase in ambient CO concentration. However, more robust studies are needed to confirm the actual association between air pollution and myocardial infarction.

Evidence examining the association between air pollution and acute decompensation of heart failure^20^ showed that hospitalization or death was related to an increase in CO (3.52% per 1 part per million; 95% CI 2.52 to 4.54), SO_2_(2.36% per 10 parts per billion; 95% CI 1.35 to 3.38) and NO_2_(1.70% per 10 parts per billion; 95% CI 1.25 to 2.16). Particle concentrations were associated with hospitalization due to heart failure or death (PM_2.5_: 2.12% per 10 μg/m^3^ , 95% CI 1.42 to 2.82; PM_10_: 1.63% per 10 μg/m^3^ , 95% CI 1.20 to 2.07). Namely, they were stronger when observed on the day of exposure, and the effects were more persistent for PM_2.5_.

The relationship between maternal exposure to air pollution and the risk of congenital heart disease in offspring has also been studied. In a meta-analysis conducted by Hu et al.,^21^ CO exposure was associated with an increased risk of tetralogy of Fallot (OR 1.21, 95% CI 1.04 to 1.41). Elevated risk of atrial septal defect was found for each 10-mg/m^3^ increment, as well as for each increase of 10 ppb in PM_10_and O_3_exposure, respectively (OR 1.04, 95% CI 1.00 to 1.09; and OR 1.09, 95% CI 1.02 to 1.17). In turn, categorical NO_2_exposure was associated with an increased risk of aortic coarctation (OR for high versus low 1.14, 95% CI 1.02 to 1.26).

Chowdhury et al.,^22^ when evaluating the association between metal pollution and cardiovascular outcomes, showed that exposure to arsenic, lead, cadmium, and copper was associated with an increased risk of CVD, especially coronary artery disease. There was a linear dose-response relationship for arsenic, lead, and cadmium with cardiovascular outcomes.

The PURE study^23^ evaluated the association of 14 possible modifiable risk factors with cardiovascular mortality, carried out in 21 countries on 5 continents, observing levels of education and income. Air pollution was associated with a higher proportion of CVD and deaths in low-income countries. In low- and middle-income countries, household air pollution, poor diet, and low education had a stronger effect on cardiovascular death than in high-income countries. According to the PURE study, a perverse logic is maintained: rich countries pollute, and poor countries get sick and die.

An important piece of information to be discussed concerns the impact of a possible reduction in global mortality attributed to pollution. WHO data describe that pollution due to annual exposure to PM_2.5_levels of 10 µg/m^3^ , when applying the new recommendation put forth in the 2021 WHO Guideline^24^ for permissible levels of exposure to PM_2.5_of 5 µg/m^3^ , mortality could be reduced by 79.5%. It should be noted that, in 2019, according to the Institute for Health Metrics and Evaluation, air pollution ranked fourth as a modifiable risk factor, after high blood pressure, tobacco use, and inappropriate diet.^3^

We are also facing extreme climate changes (floods, fires, and large areas of melting ice), which are also directly related to pollution, in addition to other factors that increase global warming, directly and/or indirectly impacting the global health of the planet. These changes are happening with an average warming of just 1.1 °C in relation to pre-industrial levels. The most recent Intergovernmental Panel on Climate Change (IPCC)^25^ report concludes that this is merely a preview of things to come. The report shows that the world is likely to reach or exceed 1.5 °C of warming over the next 2 decades—sooner than previously thought. Therefore, limiting warming to this level and avoiding the most severe climate impacts depends on actions during this decade. Only ambitious emission cuts will keep global temperature rise to 1.5 °C, the limit scientists say is needed to prevent major climate impacts. In a scenario of high emissions, the IPCC has stated that the world could warm much more, reaching an apocalyptic 5.7 °C by 2100.

In moving toward the conclusion and changing the tone slightly, leaving the destructive behind and looking toward the constructive, in the Northeast Region of Brazil and certainly in many other places in our country, there are movements in favor of the health of nature. And this is what is happening in the small and traditional community of Jacarapé, located on the coast of Paraíba. Based on the initiative of the first author of this article, in partnership with several members of the community, environmental education practices are being developed extensively. Based out of the small Public Health Clinic ( Figure 1 ), the relationship between environmental pollution and cardiovascular risk has been widely discussed. Education about environmental pollution is carried out through lectures with patients, other members of the community, school groups in the region, and visitors. In addition, an Environmental Observatory is being constructed in Jacarapé, in partnership with the Federal University of Paraíba (UFPB). At this location, sustainable policies for basic sanitation are being implanted, in addition to the replanting of 5000 mangrove seedlings and monitoring of the water quality of the Jacarapé and Cuiá Rivers. These practices, in their due proportions of the microcosm, are in line with the words of the former president of the American College of Cardiology Richard J. Kovacs: “Clinicians have a responsibility to educate their patients, their colleagues and their communities at large on the connection between air pollution and CVD risk”.^3^

**Figure 1:** – Public health clinic in the small, traditional community of Jacarapé, Paraíba, Brazil.

Finally, to face this scenario of exponential degradation of the environment, it is also up to us cardiologists to call on the general population and especially health workers, for actions that dramatically reduce the destruction of nature. Acting against pollution is, above all, an act of citizenship, an act that is non-partisan and that benefits those who vote for the most different political parties or cheer for any number of football teams throughout Brazil and worldwide, or be it, all of us. Therefore, to face the exponential degradation of this place where more than 8 billion inhabitants live (among many other living beings), the song by the poet from Paraíba, Geraldo Vandré, fits like a glove: “Those who know choose the time. They do not wait for it to happen.”
